# How to recognize and respond to viral re-positivity and disease relapse in patients with COVID-19

**DOI:** 10.7189/jogh.11.03043

**Published:** 2021-03-01

**Authors:** Shiliang Zheng, Caide Liu, Yuhua Chi, Xiaodong Sun

**Affiliations:** 1 Department of General medicine, Affiliated Hospital of Weifang Medical University, Weifang, China; 2 Department of Endocrinology, Affiliated Hospital of Weifang Medical University, Weifang, China; 3 Clinical Research Center, Affiliated Hospital of Weifang Medical University, Weifang, China

Coronavirus disease (COVID-19) in December 2019 has spread faster than anyone expected. As of September 1, there have been more than 26 million confirmed cases and 800 000 deaths worldwide in 2020. Currently, a large number of patients are cured and discharged from hospitals and have been found to test positive again for viral nucleic acid [1]. An early report that approximately 14% of discharged patients in Guangdong Province of China underwent a positive nucleic acid retest [2], causing confusion and concern among clinicians. Since then, there have been cases of the reappearance of SARS-COV-2 positive within a longer period after hospital discharge [3,4]. On August 28, 2020, Hong Kong scholars confirmed by whole-genome sequencing that a 33-year-old male patient who recovered from COVID-19 infection 142 days earlier was infected with SARS-COV-2 for the second time, rather than continuing to contract the virus after the first infection [5]. To explore the re-emergence and recurrence of COVID-19, we reviewed the literature and proposed strategies to address the concerns and confusion of the public and physicians about this situation, and comprehensive management and prevention of COVID-19 will help to continue to fully control the epidemic.

## COVID-19 FEATURES

SARS-COV-2 is a novel coronavirus belonging to the β genus, including five basic genes that target four structural proteins (N, E, M and S) and RNA-dependent RNA polymerase (RdRp), which caused COVID-19 and to which the population is generally susceptible. Severe Acute Respiratory Syndrome Coronavirus 2(SARS-CoV-2) is highly contagious and spreads rapidly, mainly through respiratory droplets and close contact, but can also be caused by contact with virus-contaminated objects [6,7]. The general presentation is fever, dry cough, and fatigue. Some patients presented with decreased or lost sense of smell or taste as the first symptom and a few had nasal congestion, runny nose, sore throat, conjunctivitis, myalgia and diarrhea. In severe cases, dyspnea and/or hypoxemia develops most often one week after the onset of symptoms, rapidly progressing to acute respiratory distress syndrome, septic shock, uncorrectable metabolic acidosis and coagulopathy, and multiple organ failure [8,9]. Most patients with COVID-19 have a good prognosis, with a few in critical condition, most often in the elderly and in patients with chronic underlying diseases.

## VIRAL RE-POSITIVE AND COVID-19 RELAPSE

The gold standard for COVID-19 diagnosis is to detect SARS-CoV-2 in a patient. As patients have a dry cough and no sputum, we often use throat swabs or nose swabs to measure the virus, rather than lower respiratory secretions. This resulted in multiple negative tests for SARS-CoV-2, which delayed the diagnosis of COVID-19 [10,11]. Approximately 0.64% of human lung cells were confirmed to express angiotensin-converting enzyme 2 (ACE2), of which 83% were alveolar type 2 cells(AT2). In addition, about 1.4% of AT2 cells express ACE2 [12]. SARS-CoV-2 can enter host cells through the cell receptor ACE2, and AT2 may be a target cell of SARS-CoV-2. The virus primarily attacks the peripheral airways and alveoli of the lower respiratory tract [13], where the number and concentration of viral particles are greatest, rather than the nasopharynx of the upper respiratory tract. Nasal or pharyngeal swabs have a low positive rate of detecting viral nucleic acids, which not only does not provide timely and accurate information in the early stages of the disease, but also does not accurately detect viral residues and COVID-19 at the time of discharge from the hospital [14].

**Figure:**
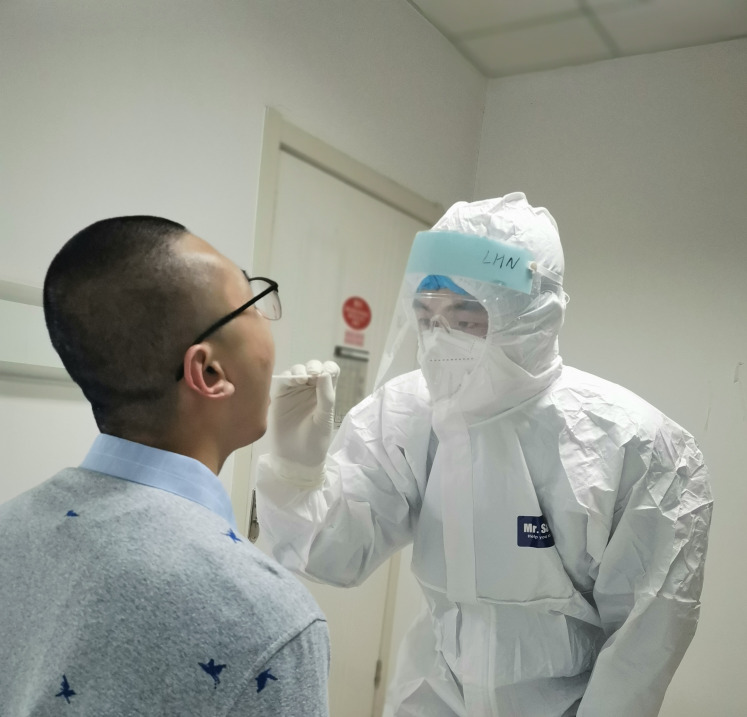
Photo: Pharyngeal swab or nasal swab is likely to be false-negative, requiring to standardize our sampling and rigorous testing practices (from Caidu Liu’s collection, used with permission).

In the early stage of COVID-19 onset in China, a large number of patients developed the disease in a short period. After aggressive treatment, many patients were discharged centrally, which may loosen the discharge criteria. As a result, about 14% of discharged patients with positive nucleic acid retest emerged in Guangdong early [2]. A cohort study of 414 patients diagnosed with COVID-19 also showed that 16.7% of COVID-19 patients tested positive for SARS-CoV-2 RNA again 1-3 times after discharge during a strict 14-day isolation period [15]. All the above cases were discharged with medication. Another single-center study reported that eight of 108 patients diagnosed with COVID-19 became SARS-CoV-2 positive and were readmitted to the hospital from February 10 to April 13, 2020 [16]. An 82-year-old man, diagnosed with COVID-19 in early April 2020 due to fever, was hospitalized for more than one month and subsequently had two negative RT-PCR for SARS-CoV-2 24 hours apart. He was discharged on the 39^th^ day of his hospitalization. Ten days after discharge, he had another fever and a positive RT-PCR for SARS-CoV-2. After another 12 days of hospitalization, he recovered and was discharged from the hospital [17]. It has also been reported that the virus remains positive for 60 days after the patient's clinical symptoms have disappeared. It has been suggested that viral clearance may be delayed depending on the patient's condition, such as hypertension, underlying diseases such as diabetes, and patients who have received glucocorticoid therapy. It has also been speculated that the positive viral RNA signal may come from a “dead” virus or viral gene fragment, but viral replication is not active [18].

To date, there is no conclusive evidence regarding the duration of self-protective antibodies produced after COVID-19 infection. Whether post-discharge viral relapses are due to viral mutation or repeated infection or even recurrence caused by infection with a different strain of virus is still questionable [19-21], but there is no evidence to confirm this question. Antibodies have also been tested for the presence of antibodies in patients with viral relapses, and it is still suspected that these relapses are infectious [22]. Until August 28, 2020, Hong Kong scholars confirmed a man who recovered from COVID-19 was infected again four-and-a-half months later using whole-genome sequencing [5]. This again raises concerns and even doubts about the efficacy of the COVID-19 vaccine that will soon enter widespread clinical use. We believe that after the COVID-19 infection, the body produces corresponding specific antibodies to fight the virus, thus protecting the body for a certain period and helping the body to resist the second infection. Therefore, the chances of recurrence within a few months for the cured patient are minimal. The infection rate of different virus strains is very low, so there is no need to worry.

## STRICTLY ENFORCE DISCHARGE CONDITIONS FOR PATIENTS WITH COVID-19

The discharge criteria for patients with COVID-19 in China are temperature return to normal for more than 3 days, significant improvement in respiratory symptoms, acute exudative lesions on lung imaging, and two consecutive negative nucleic acid tests on respiratory specimens (with a sampling interval of at least 24 hours). As the cough disappears in patients discharged with COVID-19, SARS-COV-2 with a pharyngeal swab or nasal swab was measured, which is likely to be false-negative, requiring us to look for lower respiratory specimens while standardizing our sampling and rigorous testing practices. Nebulization of induced sputum can improve the detection rate of the virus and is uncomplicated and readily accepted by patients, so the use of induced sputum for two determinations of the virus should be encouraged [14,23]. In patients with delayed discharge such as diabetes mellitus, chronic obstructive pulmonary disease, hypertension, coronary heart disease, heart failure, and renal and hepatic insufficiency, an increase in the number of nucleic acid tests may be considered, especially in elderly patients with these diseases. For patients who meet the above clinical discharge criteria, if the nucleic acid test remains consistently positive for more than 4 weeks, a comprehensive assessment of the patient's infectiousness by antibody testing and viral culture isolation is recommended.

## STRENGTHEN CENTRALIZED ISOLATION MANAGEMENT OF COVID-19 PATIENTS AFTER DISCHARGE

After patients with COVID-19 are cured and discharged from the hospital, they are transferred to a designated area for continued medical isolation and observation for 14 days. Medical institutions and relevant departments should make cooperative arrangements for discharged patients to ensure a seamless and safe handover. The isolation point should be arranged in a unique hotel or hospital isolation areas as far as possible. Each discharged patient is arranged in a well-ventilated single room, under the guidance of a doctor for self-health monitoring, wearing a mask, and the isolation room should always open windows to maintain air circulation. Shared meals, close contact with others and going out are avoided as much as possible; hand hygiene and daily cleaning are required. Daily necessities during isolation should be washed and disinfected separately.

After discharging patients from isolation for 14 days, the docking hospital should perform another pharyngeal swab and chest CT, and the patient should be released from isolation only if the novel coronavirus nucleic acid is still negative, the lung inflammatory lesion is mostly absorbed, or the fibro ligament remains. After patients are released from isolation, they should continue to monitor their health status, avoid going to crowded places, and develop the habit of “maintaining a one-meter social distance”, washing hands frequently, wearing a mask, and using communal chopsticks or sharing meals [24,25]. It is crucial to choose an appropriate respiratory rehabilitation exercise routine, to eat a diet high in protein, vitamins and calories, with plenty of fresh vegetables and fruits, to maintain a regular schedule, adequate sleep and a relaxed mood and to have reviews at 4, 12 and 24 weeks include clinical symptoms, blood tests, chest CT, IgM antibodies, IgG and nucleic acid tests.

## STANDARDIZE THE MANAGEMENT OF THE COVID-19 EPIDEMIC

In order to implement the requirements of “external prevention and internal resistance” in the normalization of epidemic prevention and control, China has implemented the normalization of the management of COVID-19 epidemics. First, we continue to strengthen hospital infection management, strictly implement fever clinics management requirements, strengthen patient admission management, strengthen ward management, develop a strict escort and visitation system, strengthen nucleic acid testing, strict implementation of standard prevention regulations, and carry out in-hospital infection risk inspection and rectification. After entering the country, foreigners are routinely quarantined in domestic hotels for 14 days without symptoms and can only return home after a negative nucleic acid test. The requirement to wear masks when entering supermarkets, hotels and other public places has also played an important role in the prevention of COVID-19.

After the control of the COVID-19 epidemic in China, there were concentrated confirmed cases in Harbin, Dalian, Beijing, and Xinjiang, and the health department swiftly paid great attention to track and medically isolate the close contacts, and to provide active treatment to the infected, which quickly quelled the epidemic. The success of epidemic prevention and control in China owed to the great attention of the government and the positive response of the people to preventive measures.

## SPECIAL GROUPS SHOULD BE VACCINATED AS EARLY AS POSSIBLE

Although vaccines have not entered the market yet, a vaccine completed a Phase II clinical trial in China in June. The Ad5-nCoV vaccine, developed by Academician Wei Chen's team and Consino Bio, adopted adenovirus vector vaccine technology, and its phase II clinical trial results showed that 99.5% of subjects developed specific antibodies, and 89.0% developed antibodies to this recombinant neo-crown vaccine at 28 days after a single vaccination. The specific cellular immune response is expected to provide a “dual protective response” against COVID-19 infection [26]. In July, three new vaccines have entered Phase III clinical trials in China, and on July 22, 2020, China officially launched the emergency use of the new coronavirus vaccine. It is mainly used for special populations such as medical personnel, epidemic prevention personnel, border guards, and those who ensure the basic operation of cities. We believe that these four groups of people should be vaccinated in advance if necessary. In addition, we believe that the old at risk of infection should also be vaccinated, as studies have shown that elderly people vaccinated also produce “sustained high levels” of neutralizing antibodies, comparable to the young [27].

In conclusion, recurrence of COVID-19 is rare, and enhanced medical isolation for 14 days after discharge can prevent transmission. The implementation of standardized management of the epidemic and early vaccination of special populations can better prevent the occurrence of COVID-19.
